# *N*‑heterocyclic carbene complexes provide a platform for functional amphidynamic crystals

**DOI:** 10.1038/s42004-021-00458-6

**Published:** 2021-02-19

**Authors:** Victoria Richards

**Affiliations:** Communications Chemistry, https://www.nature.com/commschem/

## Abstract

Organizing molecular rotors in highly ordered crystals enables the use of molecular motion to control physical properties. Now, *N*‑heterocyclic carbene complexes form a basis for molecular gyroscopes in which solid-state emission is quenched by axle rotation.

Incorporating rotational elements into crystals provides the opportunity to use molecular motion to tune material properties. Solid-state emission can be controlled in amphidynamic crystals—those that accommodate 2-fold rotational motion—through rotation-induced luminescence quenching^1–3^. Designing systems with this functionality, however, remains a challenge. Now, Mingoo Jin, Hajime Ito, and colleagues from Hokkaido University, Japan and University of California Los Angeles, USA, construct molecular gyroscopes composed of binuclear *N*‑heterocyclic carbene complexes, in which rotary behavior and optical properties are fine-tuned by the nature of the transition metal ions that link the rotator unit to the static units (10.1021/jacs.0c11981)^4^.

The team envisioned that bulky, concave *N*‑heterocyclic carbene (NHC) ligands could shield a coaxially coordinated central pyrazine rotator (Fig. 1), thereby generating sufficient volume around the rotating unit for large angular displacements. They additionally conceived that the nature of the metal ion could modulate the rotary motion. Indeed, they found that the Au(I) rotor possesses an activation energy of 3.4 kcal/mol lower than that of the Cu(I) rotor, in spite of the complexes adopting very similar overall packing structures. Steric and electronic effects are found to be responsible for this difference in the rotational energy barrier. The larger Au(I) ions dictate a 0.2 Å increase in the length of the rotation axis, resulting in less steric repulsion between the NHC ligands and the pyrazine rotator. In addition, greater π-back-donation from the Au(I) metal ions to the rotator, owing to increased *d* and π* orbital overlap in comparison with the Cu(I) complex, is shown to stabilize the transition state.

**Fig. 1:**
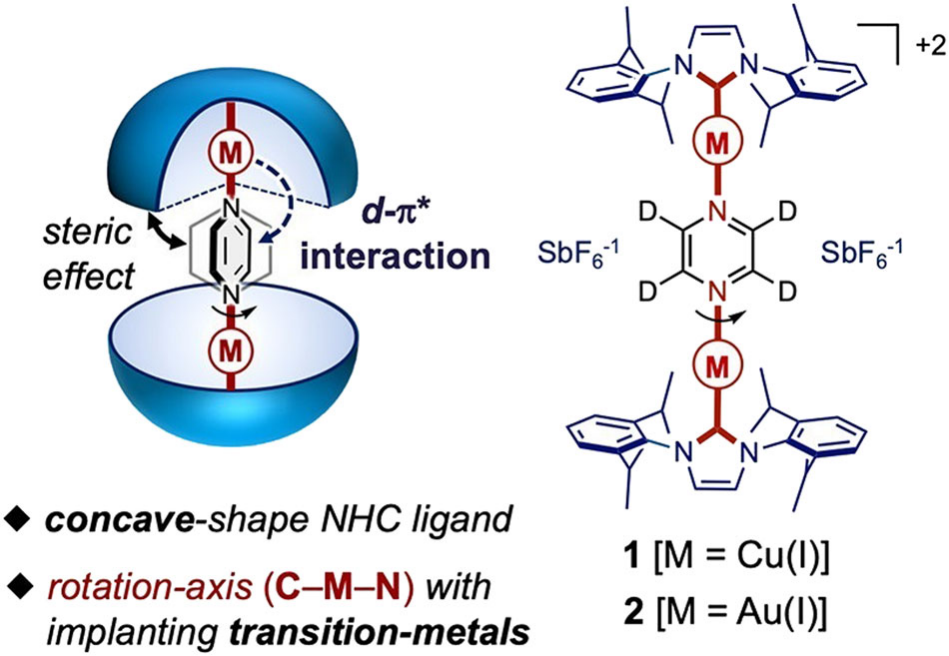
*N*‑heterocyclic carbene complexes serve as molecular gyroscopes. Binuclear Cu(I) and Au(I) complexes of 1,3-bis(2,6-diisopropylphenyl)imidazol-2-ylidene ligands bridged by deuterated pyrazine provide a scaffold for 2-fold rotational motion in luminescent amphidynamic crystals. Adapted with permission from *J. Am. Chem. Soc*. **139**, 18115– 18121. Copyright (2021) American Chemical Society.

The emission properties are also tuned by switching the metal ion. While the Au(I) rotor exhibits blue luminescence, greater electronic delocalization in the Cu(I) complex causes a distinct red-shift in the solid-state emission. In both cases, molecular rotation is found to induce emission quenching.

Thanks to the ease of synthesis of these gyroscopic rotors, and considering the vast library of NHC ligands that organometallic chemists have designed to date, it isn’t difficult to imagine that this system could serve as a platform for the construction of a range of tunable amphidynamic crystals. “Chiral NHC ligands possessing asymmetric structural moieties may allow for the design and formation of chiral space in the crystalline phase, which could contribute to exploring non-linear photophysical properties in the solid-state.”, comments Mingoo Jin.
